# Musculoskeletal application and validation of speckle-tracking ultrasonography

**DOI:** 10.1186/s12891-019-2562-8

**Published:** 2019-05-04

**Authors:** Lars Henrik Frich, Kate Lykke Lambertsen, John Hjarbaek, Jordi Sanchez Dahl, Anders Holsgaard-Larsen

**Affiliations:** 1 Department of Orthopaedics and Traumatology, Odense University Hospital, J.B. Winsloewsvej 4, 5000 Odense, Denmark; 2 Orthopaedic research unit, University of Southern Denmark, J.B. Winsloewsvej 4, 5000 Odense, Denmark; 3 OPEN, Odense Patient data Explorative Network, Odense University Hospital/Department of Clinical Research, University of Southern Denmark, J.B. Winsloewsvej 4, 5000 Odense, Denmark; 4 Department of Neurobiology Research, Institute of Molecular Medicine, University of Southern Denmark, Odense, Denmark; 5 Department of Neurology, Odense University Hospital, Odense, Denmark; 6 BRIDGE - Brain Research - Inter-Disciplinary Guided Excellence, Department of Clinical Research, University of Southern Denmark, Odense, Denmark; 7 Department of Radiology, Odense University Hospital, Odense, Denmark; 8 Department of Cardiology, Odense University Hospital, Odense, Denmark

**Keywords:** Speckle tracking, Ultrasonography, Strain, Skeletal muscle, Non-invasive

## Abstract

**Background:**

Diseased, injured, or dysfunctional skeletal muscles may demonstrate abnormal function and contractility. Currently, only few in vivo imaging techniques are able to characterize the contractile properties of muscle tissue. This study aimed to test the hypothesis that muscle strain can be tracked in two upper extremity skeletal muscles by speckle-tracking ultrasonography (STU) and correlates with isometric muscle contractions.

**Methods:**

A convenience sample of 10 healthy, adult volunteers with normal shoulder function were tested. The 5 women and 5 men had a mean age of 45 years (range: 39–59 years) and BMI < 30. STU was applied to the supraspinatus (SS) and biceps brachii (BB) muscles using a M11 L-MHz linear transducer (frequency 8–15 MHz) hooked to a Vivid E 9TM ultrasound machine. Strain validation was performed by correlating peak strain against standardized sub-maximal, isometric load conditions of the two muscles (20–80% of maximal voluntary contraction) using a custom-built muscle dynamometer based on strain-gauge technique. Data were analyzed offline using the EchoPac speckle-tracking software and were blinded to the examiner.

**Results:**

Intramuscular strain measured by STU in the SS and BB muscles showed moderate to strong correlations with external muscle load (SS: r = − 0.76, *p* < 0.0001 and BB: r = − 0.60, *p* < 0.0001). We found strain to vary from approximately 10–20% during increasing submaximal, isometric conditions.

**Conclusions:**

We demonstrate that STU can be applied on healthy skeletal musculature (SS and BB muscles). The observed correlations between strain and isometric contractions suggest a valid technique. However, the concept of measuring muscle strain non-invasively needs further investigation for validity, accuracy, responsiveness, and reliability before its therapeutic and research potential can be realized.

## Background

Muscle strain is the result of tension-generating sites within muscle fibers acting cumulatively to generate contraction [1]. Skeletal musculature contains bundles of muscle fibers called myofibrils, and each myofibril is a chain of sarcomeres, which are the smallest repeating functional units in the muscle. Each sarcomere is composed of long, fibrous proteins that slide past each other when a muscle contracts or relaxes [2]. In skeletal muscles, contraction is stimulated by action potentials transmitted by motor neurons. To date, electromyography is used as a pseudo-outcome for the assessment of muscle performance by measuring the sum of active motor units in the vicinity of the electrodes. Electromyography may be biased, however, and it is not a direct measure of muscle strain [3]. While attempts have been made to assess muscle performance indirectly using muscle elasticity [4] or muscle thickness [5] as proxies for muscle contractility, such measurements have failed to demonstrate value in the diagnosis of muscle dysfunction and in supporting diagnostic and therapeutic decisions [6].

Currently, few in vivo imaging techniques are able to characterize the contractile properties of muscle tissue. Among these, modern ultrasound apparature equipped with automatic tracking of clusters of speckles has been used for functional assessment of the heart [7] and the diaphragm [8]. Strain analysis is being embraced and increasingly adopted in many echocardiography laboratories worldwide [9] and was recently applied in deep dorsal neck muscles in individuals with whiplash-associated disorders [10, 11]. Altered strain outputs have been suggested between diseased and healthy neck muscles, [10] and there is some evidence of a relationship between strain and external force output in the biceps brachii (BB) muscle [12]. It remains unclear, however, whether muscle strain values in different muscles are associated with the intensity of voluntary muscle contractions. The overall aim of this study was therefore to address the clinical and technological research gap within the field of in vivo muscle contraction by assessing the validity of speckle technology as a non-invasive, clinical measure of muscle strain. Our specific objective was to test the hypotheses that displacement of the speckle pattern (representing muscle strain) can be tracked in two upper extremity skeletal muscles speckle tracking ultrasonography, and that this displacement correlates with isometric muscle contractions.

## Methods

### Subjects

This study was performed at Odense University Hospital, Odense, Denmark from May to December 2017. A convenience sample of ten healthy, adult volunteers with normal shoulder function participated in the study and underwent STU. The five women and five men had a mean age of 45 years (range: 39–59 years) and body mass index < 30. None of them had a history of neuromuscular disorder, and each gave written informed consent prior to participation in the study. The supraspinatus (SS) muscle of the right shoulder and the BB muscle of the right arm were chosen for measurements. The number of subjects (*n* = 10) was chosen based on prior pilot-testing (see below).

The research was conducted in accordance with the Helsinki II Declaration. The study was approved by the Danish Biomedical Research Ethical Committee for the Region of Southern Denmark (Permission No: S-20160037) and reported to the Data Inspectorate (J. No.: 16/9714). The paper follows the “STROBE Statement” guidelines for reporting observational studies [13].

### Maximal voluntary contractions (MVC) testing protocol

Voluntary muscle contractions were performed under controlled conditions to measure the external force using custom-made dynamometry (Force Transducer, U9C, ≥2kN, Hottinger Baldwin Messtechnik GmbH, Darmstadt, Germany). The subject was seated comfortably, and the arm was supported and strapped to the muscle dynamometer that was connected to a computer via a combined Wheatstone bridge, 24 bit analog-to-digital converter (cDAQ-9174/NI-9237, National Instruments, Austin, TX, USA) with custom-developed software (Labview version 2017, National Instruments). The force transducer signals were converted by linear regression into Newton (N), with real-time visual feedback on a PC monitor.

Before testing, a standardized warm-up routine for the shoulder and arm was carried out. The motion pattern for the tests was determined by the most isolated unidirectional flow of the fibers in the BB and SS muscles that were visualized on the screen of the ultrasound scanner. Tests of the SS muscle were therefore performed with the elbow flexed to 90 degrees and the shoulder at 20 degrees of elevation in the scapular plane. The BB muscle was tested with the elbow flexed to 90 degrees and the forearm in full supination.

Three MVC were performed for the SS and for the BB. These MVC tests were continued for five seconds and conducted at two-minute intervals. The peak force signal (F_max_) was determined for each MVC, and the contraction with the highest F_max_ was selected for determining submaximal test contractions at 20–80% for the subsequent strain measurements.

### Speckle-tracking ultrasonography (STU)

Skeletal ultrasound was performed using a M11 L-MHz linear transducer (frequency 8–15 MHz) with a Vivid E 9TM ultrasound machine (General Electric Healthcare, Horten, Norway). Images were acquired at > 140 fps, and the mm-pixel ratio was 0.062 mm/px.

**Fig. 1:**
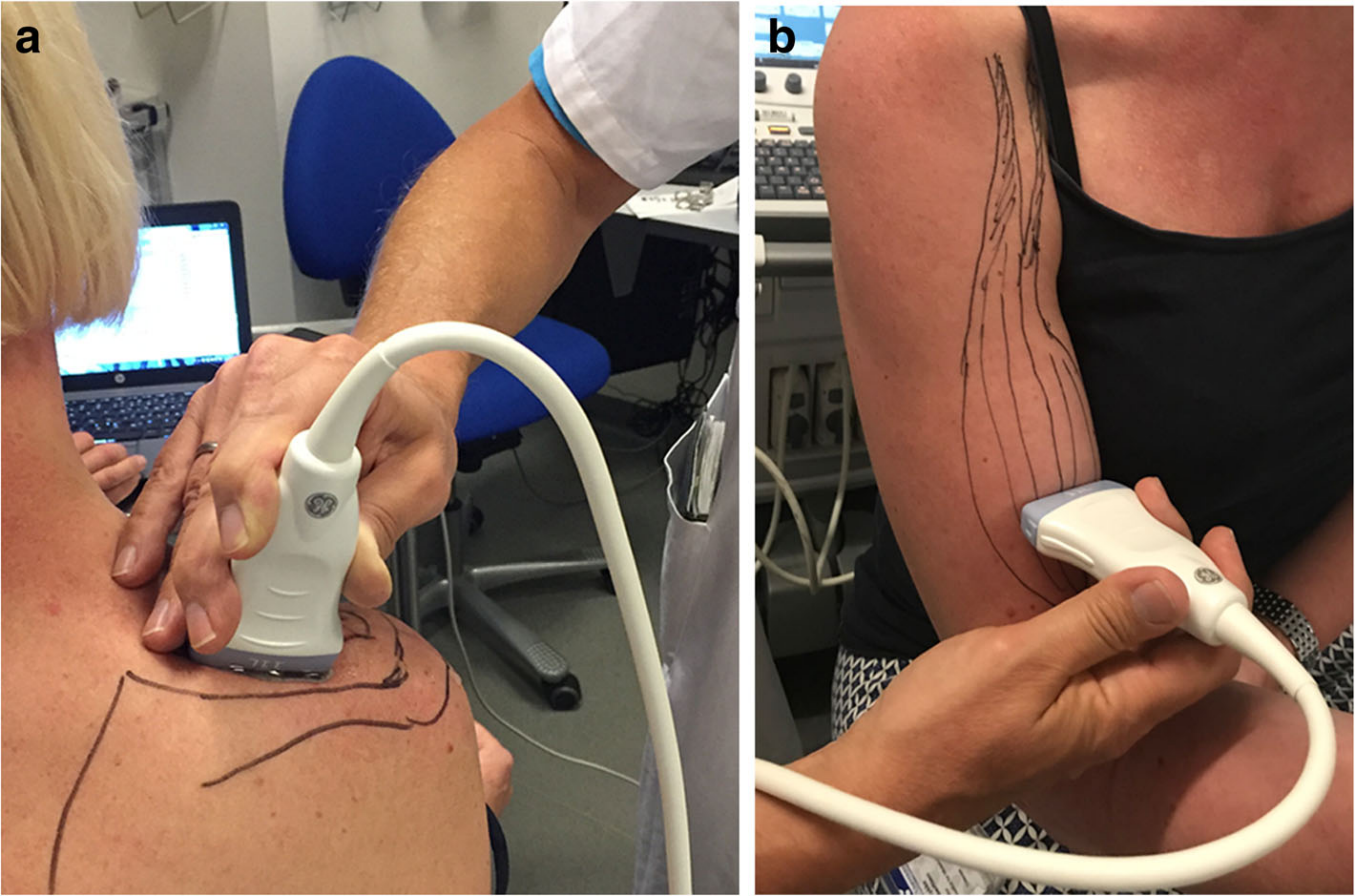
2D speckle ultrasound set-up. **a**, **b**, Transducer position to visualize the supraspinatus (**a**) and biceps (**b**) muscles

As described above, STU analysis was performed with the patient in a stable sitting position and strapped to the muscle dynamometer. Anatomical landmarks of the shoulder were identified and marked with pencil. The integrity of tendon insertion of the SS was checked. For imaging of the SS muscle, the transducer was placed over the SS fossa (Fig. 1a). The deep elements of the SS muscle were identified, and STU was recorded just lateral to the scapular spine. For imaging of the BB, the transducer was placed over the muscle belly of the long head of the biceps and at a level approximately 10 cm below the coracoid process (Fig. 1b). The transducer was in both scenarios oriented parallel to the loading axis of the muscles to overcome influence of pennation of the muscle fibers.

The ultrasound probe was kept in the same position relative to the skin during recordings of voluntary muscle contractions under load. The submaximal target force (%MVC), together with the actual force trajectory, applied by the subject was visible in the form of a bio-feedback curve displayed in real-time on a PC monitor.

A custom-made, manual switch giving electrical signals through the echocardiographic input to the ultrasound machine was applied to ensure synchronization between the muscle force signals and the ultrasound recordings.

At the onset of the experiment, the subject was asked to perform isolated voluntary isometric contraction cycles aiming at 40, 60, and 80% of MVC of the SS muscle and aiming at 20, 40, and 60% of MVC of the BB. A series of four individual cycles at each intensity level was performed with a duration of 3 s and a 2-min break between contractions. Post-examination, the actual applied force (slightly above or below target force) for each individual in each test cycle was determined as a percentage of the MVC.

Pilot-testing demonstrated that valid STU of the BB required a pre-tensioning of approximately 5% MVC to counteract slackness of the muscle-tendon complex, thus keeping speckle displacement within the measuring region of interest (ROI). As the current set-up for stabilizing the test person, the ultrasound transducer, and the muscle dynamometer did not allow measurements at or above 80% for the BB, the STU was restricted to target forces between 20 and 60% for this muscle.

### Muscle strain analysis

Strain is a unit-less measurement of dimensional tissue change and a measure of muscle contractility [14]. Strain was analyzed offline, blinded to the examiner and was tracked using a multi-kernel block-matching scheme devised specifically for tracking muscle movement. In practice, we used the Q-analysis function of the EchoPac speckle-tracking software developed by GE Healthcare (version BT 12). The software and technique utilized in our study have been extensively studied in the field of cardiology [15] and is today recognized as an important clinical tool and used on a daily basis. In theory, the software detects reflected scattered signals (speckles) within muscle tissue. Based on the unique movement of these speckles, it is possible to calculate muscle strain as the absolute shortening between two speckles divided by the distance between the speckles. The EchoPac speckle-tracking software, provided by GE Medical, automatically detects speckle movement.

To analyze loops (i.e. video-recorded muscle contraction), an elongated tracking ROI was manually placed along the central raphe within the muscle, and frame-to-frame displacement was estimated using multiple overlapping small kernels. The quality of the recorded loops varied in some volunteers, depending on the external load and the general movement of the arm, especially in high-loading contractions.

A built-in software quality assurance implied that low-quality loops were discarded from the analysis if i) displacement of the total muscle was larger than the image size, ii) if the probe was moved relative to the muscle, resulting in defined speckles sliding out of the ROI, or iii) if tracking was not possible in at least two of the three zones of the ROI panels. Repetitive testing resulted in only a few loops being discarded due to twisting of muscle bundles and interframe disappearance of a few speckles, which is known to happen within the kernel. The number of loops tested is listed in Table 1. The number of discarded loops did not significantly compromise the quality of the tracking.

**Table 1:** Results of the submaximal isometric contractions

Muscle (% of MVC)	Mean ± s.d.	N^0^ of loops	95% CI of mean	*P*-values
Supraspinatus				
40%	−10.12 ± 3.24	30	−11.33 to −8.91	***p < 0.001
60%	−14.85 ± 4.69	32	−16.54 to −13.16	****p < 0.0001
80%	−20.96 ± 6.30	25	−23.56 to −18.37	****p < 0.0001
Biceps brachii				
20%	−9.35 ± 4.64	18	−11.66 to −7.04	**p < 0.01
40%	−15.08 ± 5.60	23	−17.50 to −12.66	****p < 0.0001
60%	−17.77 ± 4.26	24	−19.56 to −15.97	n.s.

*MVC* maximal voluntary contractions, *s.d* standard deviation, *CI* confidence interval

For statistical comparison please refer to Fig. 3b and Fig. 4b

Statistical analysis was performed using Prism 5 software for Windows (GraphPad). Ordinary one-way ANOVA followed Tukey’s multiple comparisons test was used to analyze changes in strain with increasing contraction. An increase in strain refers to a more negative value of strain, and thus an increased shortening of the muscle.

Bartlett’s and Brown-Forsythe tests were used to test the equality of group variances. Validity was evaluated using simple linear regression with muscle strain as the dependent variable and isometric muscle force as the independent variable. The correlation coefficients were interpreted according to Dancey and Reidy [16], where an r-value of 0.1–0.3 was regarded as weak; >0.3–0.6 as moderate; >0.6–0.9 as strong; and 1 as perfect correlation. Data are presented as mean ± SD, and the significance level was set to 0.05 (5%).

## Results

**Fig. 2:**
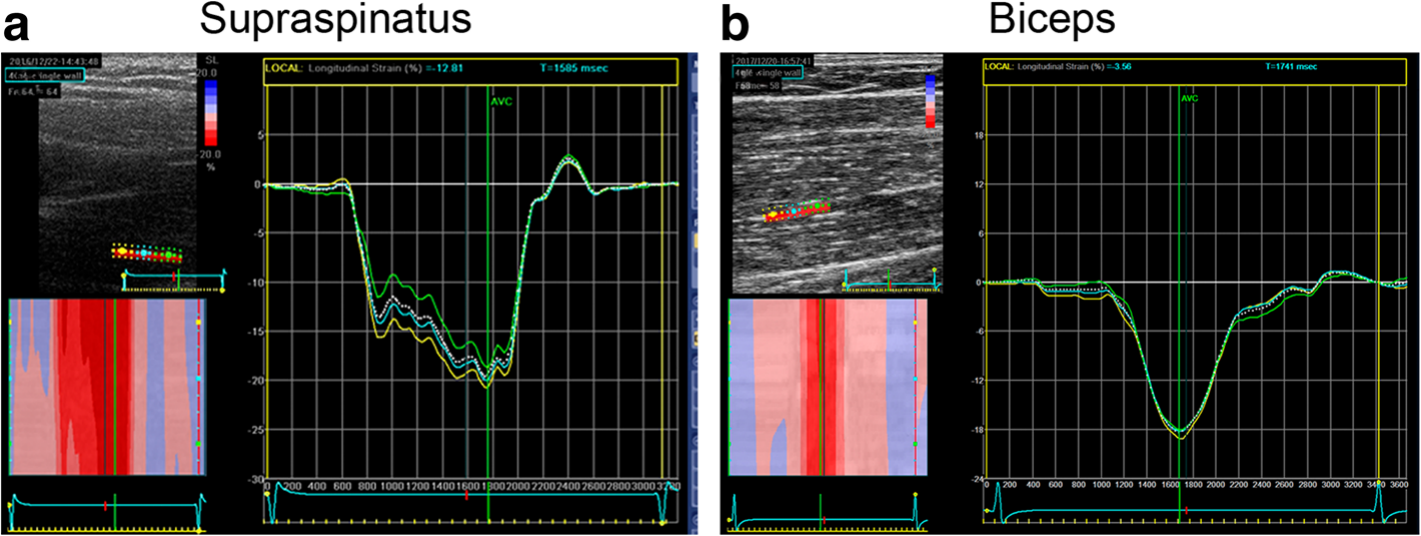
Supraspinatus and biceps muscle dynamics. **a**, **b**, Graphic depiction of speckle-tracking ultrasonographic analysis of supraspinatus (**a**) and biceps (**b**) showing representative measurements of longitudinal strain at 80 and 40% max load, respectively

The SS muscle (Fig. 2a) and the BB muscle (Fig. 2b) both showed strain patterns that could be accepted and analyzed using Q-analysis and EchoPac speckle-tracking software according to the methods described above.

**Fig. 3:**
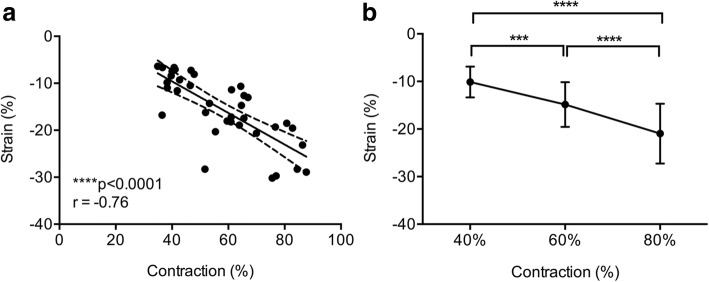
Results of 2D longitudinal supraspinatus muscle strain. **a**, Muscle strain (%) correlated negatively with increasing load (%) (95% CI: -0.87% to − 0.58%; Pearson correlation coefficient). **b**, The mean muscle strain of the normal supraspinatus muscle of ten subjects at different isometric load conditions (40, 60, and 80%) demonstrating significantly increased strain rate with increasing load condition (One-way ANOVA *p* < 0.0001, F_(2,84)_ = 34.7). Error bars represent s.d. (*n* = 10, four independent measurements/load condition). ***p < 0.001; **** *p* < 0.0001

**Fig. 4:**
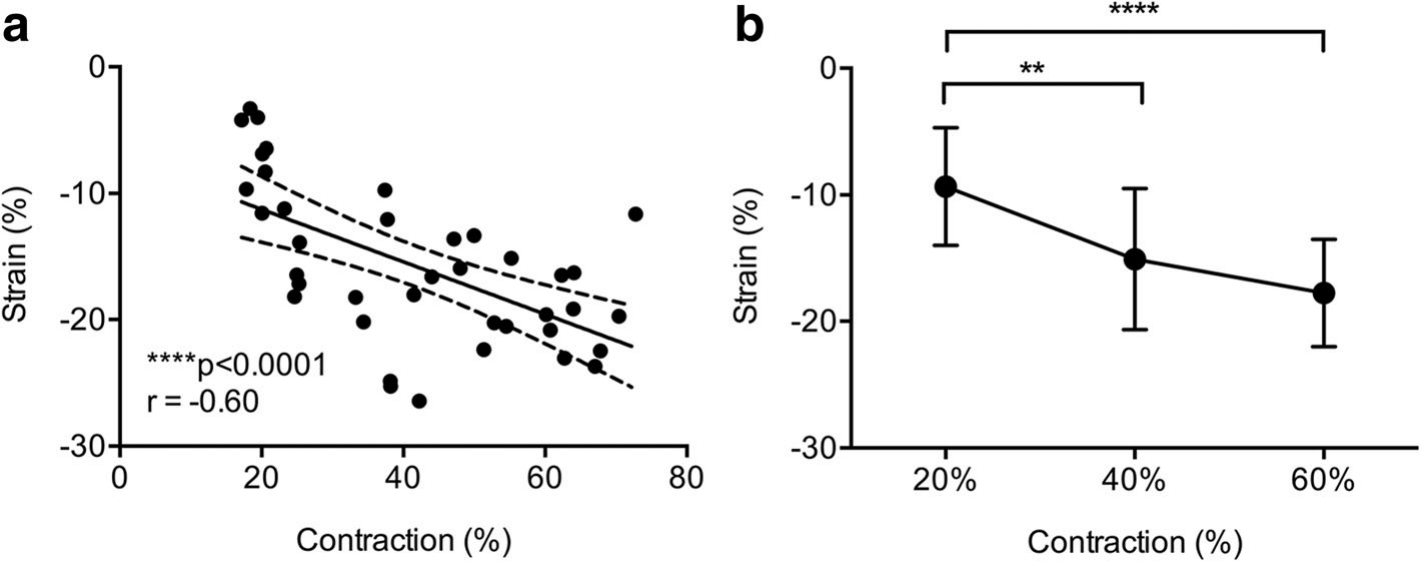
Results of 2D longitudinal biceps muscle strain. **a**, Muscle strain (%) correlated negatively with increasing load (%) (95% CI: -0.77% to − 0.35%; Pearson correlation coefficient). **b**, Strain analysis of the normal biceps muscle of ten subjects at different concentric load conditions (20, 40, and 60%) demonstrating increased strain rate with increasing load condition (One-way ANOVA *p* < 0.0001, F_(2,62)_ = 15.62). Error bars represent s.d. (*n* = 10, four independent measurements/load condition). **p < 0.01; **** *p* < 0.0001

Linear regression between the SS muscle strain and external load parameters demonstrated strong correlations (r = − 0.76, *p* < 0.0001) (Fig. 3a). The correlation between strain (%) and external load conditions of the BB muscle was moderate (r = − 0.60, *p* < 0001) (Fig. 4b).

We also found that strain in the healthy SS and BB muscles varied depending on the external muscle load contraction (*p* < 0.0001) (Figs. 3b and 4b). Thus, the mean strain was found to increase with increasing load (Table 1).

## Discussion

Speckle-tracking ultrasonography (STU) revealed intramuscular strain variations of approximately 10–20% during varying sub-maximal isometric loading of the supraspinatus (SS) and biceps (BB) muscles. Based on the findings of moderate to strong correlations between strain and isometric muscle contractions, this study provides the first steps towards validating and advancing speckle technology as a promising clinical tool for measuring skeletal muscle function.

The proposed modality is non-invasive and provides information about the strain of the human skeletal muscle during isometric contractions in healthy individuals. Other methods have been considered valid instruments for muscle testing in the past decades and are often used as proxies for muscle activity. Electromyography remains a gold standard for assessment of motor units in muscles, [3] and isometric and isokinetic dynamometry have been used for the past 40 years in the clinical setting for measuring muscle strength [17]. Isometric dynamometry is a different construct from muscle strain, but it is reliable and still qualifies as a reference for the present validation analyses [18].

In the present study, we measured muscle strain features during isometric contraction of the SS and BB muscles by applying STU. The BB is the primary mover of elbow flexion and is easily accessible for STU due to its superficial position directly beneath the skin. The SS is anatomically different from the BB and is deeper under the skin surface. The muscle is also easily accessible to STU, however, and the muscle is of interest in a clinical setting due to the well-described relationship between tendon disease and related degenerative muscle changes [19]. Greater twisting of muscle bundles is expected during increased loads, and excessive lateral displacement of speckles beyond the region of interest (ROI) may make it difficult to track the speckles in the contracting muscle. Consequently, it was only possible to achieve valid STU measures of intensity up to 60% maximal voluntary contractions (MVC) for BB and up to 80% for SS.

The current data support the hypothesis that ultrasonic speckle patterns can be tracked in vivo in human skeletal muscle and that measured muscle strain values correlate with sub-maximal isometric muscle contractions. Lopata et al [12]. demonstrated increasing strain values in relation to external force outputs of the BB in five healthy individuals using a former ultrasonography technology that had approximately half the sampling frequency (38-50 Hz) as the current technology (>140 Hz). Rahnama et al. [10] recently used custom-made software to process the unique speckle pattern formed by the acoustic ultrasound waves that were scattered and reflected upon hitting the muscles. They reported strain values for healthy dorsal neck muscles (11–34%) that were very similar to those in the current study. Our study is the first step to validate this novel technique in human skeletal muscles. Despite the lack of a gold standard, criterion validity can be tested under reasonable conditions using a widely accepted reference standard from another construct of outcome measures [20]. The current study is not a full validation of STU, however, nor does it confirm that ultrasonic speckle patterns provide accurate measures of strain.

A recent study on human Achilles tendons used STU to investigate intratendinous displacement following surgery for unilateral tendon rupture [21]. Implanted tantalum beads were used to follow displacement of tendon speckles, but this was not feasible in the present study. Within-session reliability of STU was demonstrated in a study of deep dorsal neck muscles, but this did not clearly define whether the reliability of strain parameters was calculated within or between test sessions [22]. Finally, differences in strain between individuals with whiplash-associated disorders and healthy controls have been shown [10]. However, the study did not provide evidence of an association between strain parameters and varying external loading.

Other researchers have tried various non-invasive approaches to measure muscle activity. Historically, ultrasonography has been used to measure muscle architectural parameters such as muscle thickness, [23] muscle pennation angle, [24] muscle fiber length, and muscle cross-sectional area as indirect expressions of muscle activity [25, 26]. Indirect estimates of muscle tissue properties can be made using magnetic resonance elastrography (MRE) [27, 28] and shear wave elastrography (SWE) [6]. MRE is not able to specify muscle tension, however, and the modality is hampered by being physically inappropriate. The clinical significance of SWE for mechanical properties of the tissues is still poorly understood [29]. Thickness and elasticity measurements also failed to demonstrate value in the diagnosis of muscle dysfunction and in supporting diagnostic and therapeutic decisions. This is due to difficulties in controlling the probe pressure to the skin to create linear strain curves, and because only a semi-quantitative approach can be used to determine the so-called strain ratio or to create a qualitative visual assessment with patterns, scores, or grades [6]. STU measurements are less dependent on probe pressure to the skin and can quantify muscle strain. Muscle impairment is a prevalent and major problem, and as the non-invasive STU modality is a widespread healthcare technology available throughout the world, it may enable us to monitor the treatment of patients with muscle impairment and thus revolutionize the rehabilitation of tendo-muscular damage. STU may also prove relevant for measuring the impact of exercise-induced muscle damage on performance test outcome in elite players and be a valuable tool for further studies focusing on healing processes or therapeutic interventions. Furthermore, it may potentially be useful for tracking improvements in muscle contractility and responsiveness to medical treatment in a variety of neuromuscular diseases (e.g. multiple sclerosis, muscular dystrophy, Parkinson’s disease, etc.). However, clinimetric properties of STU such as its reliability and responsiveness to treatment effects need to be established in future prospective trials.

### Limitations

A current limitation of STU is the limited capacity of the ultrasound probes that leads to difficulties with handling large strains in large muscles, such as the quadriceps. We found that if the total muscle displacement was larger than the image size or if the probe was moved relative to the muscle, the tracked speckles slid out of the ROI. This limitation may be especially pronounced when muscles are tested close to MVC and/or when slack exists in the tendon-muscle complex from resting to contracting mode. Pilot-testing demonstrated that pre-tensioning of the BB is needed to avoid excessive slack and sliding of the muscle out of probe focus. A further limitation is that the software calculations are based on algorithms patented by GE and not open to the public. In the absence of a ‘gold standard’, the current criterion validation was performed using a widely accepted reference standard from a very similar and most probably dependent validated construct (i.e. isometric muscle contractions). The observed correlations between muscle strain and isometric muscle force are thus indicative, but do not necessarily prove, that the methodology is accurate.

Operator experience, including careful control of the transducer’s position and orientation along muscle fibers, may influence the quality of the recorded loops. Furthermore, improved visual control of the applied force (%MVC) by the subject during testing may affect the accuracy of the external force measurements and corresponding speckle analysis. Finally, no inter- and/or intra-operator reliability was provided for the present set-up, and these should be conducted before implementing the technique in research or clinic.

## Conclusion

Speckle-tracking ultrasonography was applied on healthy supraspinatus and biceps brachii muscles and showed intramuscular strain variations from approximately 10–20% during varying sub-maximal isometric conditions. We found moderate to strong correlations between strain and isometric contractions, suggesting it to be a valid technique. However, this non-invasive approach to identifying muscle strain needs further investigation before its clinical potential can be realized.

## Abbreviations

BB: Biceps brachii
F_max_: Peak force signal
MCV: Maximal voluntary contractions
OPEN: Odense Patient Explorative Network
ROI: Region of interest
SS: Supraspinatus
STROBE: Strengthening the Reporting of Observational Studies in Epidemiology
STU: Speckle-tracking ultrasonography
